# Parathyroidectomy: still the best choice for the management of severe secondary hyperparathyroidism

**DOI:** 10.1590/2175-8239-JBN-2023-0024en

**Published:** 2023-12-01

**Authors:** Luiz Guilherme Fernandes Ramos, Daniela Del Pilar Via Reque Cortes, Luciene Machado dos Reis, Fabio Luiz de Menezes Montenegro, Sérgio Samir Arap, Marília D’Elboux Guimarães Brescia, Melani Ribeiro Custódio, Vanda Jorgetti, Rosilene Motta Elias, Rosa Maria Affonso Moysés

**Affiliations:** 1 Universidade Nove de Julho, São Paulo, SP, Brazil.; 2 Universidade de São Paulo, Faculdade de Medicina, Hospital das Clínicas, Serviço de Nefrologia, São Paulo, SP, Brazil.; 3 Universidade de São Paulo, Faculdade de Medicina, Hospital das Clínicas, Departamento de Cirurgia de Cabeça e Pescoço, São Paulo, SP, Brazil.

**Keywords:** Renal Insufficiency, Chronic, Hyperparathyroidism, Secondary, Cinacalcet, Parathyroidectomy, Kidney Transplantation, Insuficiência Renal Crônica, Hiperparatireoidismo Secundário, Cinacalcete, Paratireoidectomia, Transplante de Rim

## Abstract

**Introduction:**

Management of secondary hyperparathyroidism (SHPT) is a challenging endeavor with several factors contruibuting to treatment failure. Calcimimetic therapy has revolutionized the management of SHPT, leading to changes in indications and appropriate timing of parathyroidectomy (PTX) around the world.

**Methods:**

We compared response rates to clinical vs. surgical approaches to SHPT in patients on maintenance dialysis (CKD 5D) and in kidney transplant patients (Ktx). A retrospective analysis of the one-year follow-up findings was carried out. CKD 5D patients were divided into 3 groups according to treatment strategy: parathyroidectomy, clinical management without cinacalcet (named standard - STD) and with cinacalcet (STD + CIN). Ktx patients were divided into 3 groups: PTX, CIN (cinacalcet use), and observation (OBS).

**Results:**

In CKD 5D we found a significant parathormone (PTH) decrease in all groups. Despite all groups had a higher PTH at baseline, we identified a more pronounced reduction in the PTX group. Regarding severe SHPT, the difference among groups was evidently wider: 31%, 14% and 80% of STD, STD + CIN, and PTX groups reached adequate PTH levels, respectively (p<0.0001). Concerning the Ktx population, although the difference was not so impressive, a higher rate of success in the PTX group was also observed.

**Conclusion:**

PTX still seems to be the best treatment choice for SHPT, especially in patients with prolonged diseases in unresourceful scenarios.

## Resumo

**Introdução:**

O manejo do hiperparat-ireoidismo secundário (HPTS) é uma tarefa desafiadora com diversos fatores que contribuem para o fracasso do tratamento. A terapia calcimimética revolucionou o manejo do HPTS, levando a alterações nas indicações e no momento apropriado da paratireoidectomia (PTX) em todo o mundo.

**Métodos:**

Comparamos taxas de resposta às abordagens clínica vs. cirúrgica do HPTS em pacientes em diálise de manutenção (DRC 5D) e pacientes transplantados renais (TxR). Foi realizada uma análise retrospectiva dos achados de um ano de acompanhamento. Pacientes com DRC 5D foram divididos em 3 grupos de acordo com a estratégia de tratamento: paratireoidectomia, manejo clínico sem cinacalcete (denominado padrão - P) e com cinacalcete (P + CIN). Os pacientes com TxR foram divididos em 3 grupos: PTX, CIN (uso de cinacalcete) e observação (OBS).

**Resultados:**

Na DRC 5D, encontramos uma redução significativa do paratormônio (PTH) em todos os grupos. Apesar de todos os grupos apresentarem um PTH mais elevado no início do estudo, identificamos uma redução mais acentuada no grupo PTX. Com relação ao HPTS grave, a diferença entre os grupos foi evidentemente maior: 31%, 14% e 80% dos grupos P, P + CIN e PTX atingiram níveis adequados de PTH, respectivamente (p< 0,0001). Com relação à população TxR, embora a diferença não tenha sido tão impressionante, também foi observada uma taxa maior de sucesso no grupo PTX.

**Conclusão:**

A PTX ainda parece ser a melhor escolha de tratamento para o HPTS, especialmente em pacientes com doenças prolongadas em cenários sem recursos.

## Introduction

Chronic kidney disease mineral and bone disorder (CKD-MBD) is one of the main metabolic disorders associated with chronic kidney disease and highly responsible for the risk of cardiovascular events, fractures, and death^1,2^. The pathophysiology underlying SHPT involves a complex interplay of factors, including vitamin D deficiency, hyperphosphatemia, hypocalcemia, decreased renal and parathyroid expression of Klotho, as well as elevated fibroblast growth factor-23 (FGF-23)^3^. The intricate metabolic scenario is also modified by a variety of post-kidney transplant factors, including use of immunosuppressive drugs and degree of graft dysfunction^4^. An integrative and comprehensive therapeutic approach must target these various pathways, and the classical therapy for SHPT usually includes phosphate binders, vitamin D receptor activators (VDRAs), and dialysis adjustment.

The introduction of calcimimetics was a major advance in the treatment of SHPT^2^, with excellent results in terms of biochemical control and morbidity among patients in the US, Japan, and some European countries^5^. However, the lack of concrete data on how best to manage severe SHPT is reflected in current clinical practice guidelines that vary substantially by organization^6^.

The Brazilian population is of special interest, with a high prevalence of severe SHPT^7^, which is the result of limited access to VDRAs and calcimimetics. In addition, parathyroidectomy (PTX) is performed in only a few centers, which leads to a high number of patients with serum PTH levels above 1,000 pg/mL^7^. Therefore, reference centers for CKD-MBD therapy usually must deal with a waiting list for PTX, and nephrologists manage these patients by trying to avoid surgery. In this study, we tested the hypothesis that patients with severe SHPT have a poor response to clinical management and should be referred to PTX.

## Methods

### Source Population and Data Collection

In this retrospective cohort study, we aimed to compare the clinical vs. surgical approach to SHPT among CKD 5D (patients on maintenance dialysis) and kidney transplant (Ktx) patients from the nephrology outpatient clinic of the Hospital das Clinicas, Universidade de São Paulo, Brazil. The local ethics committee has approved the study (CAPpesq # 45163715.4.0000.0068).

**Figure 1.**
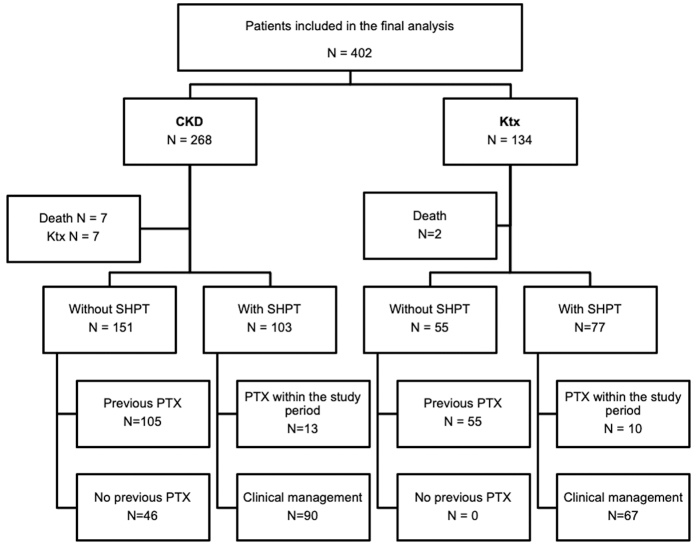
Flowchart of patient selection.

There were 402 adult patients under follow-up at the CKD-MBD clinic who had at least two visits between July 1^st^, 2017 and June 30^th^, 2018. As shown in Figure 1, patients were divided into two groups: CKD (n = 268) and KTx (n = 134). Within the CKD group, 103 had SHPT (defined as PTH > 300 pg/mL). A standard therapy that included native vitamin D, vitamin D receptor activators (VDRAs), and phosphate binders (calcium and non-calcium based) was prescribed to 28 of these patients (STD group). Cinacalcet was incorporated into STD therapy in 62 patients (STD + CIN group). PTX was performed in the remaining 13 patients (PTX group). A sub-analysis of patients with a severe HPTS, defined as baseline PTH levels > 800 pg/mL, was also performed. In the KTx group, 77 had SHPT (defined as PTH > 100 pg/mL and/or serum ionized calcium > 5.3 mg/dL). An observational therapy was applied to 31 participants (OBS group), whereas cinacalcet was prescribed to 36 (CIN group) and PTX was performed in 10 patients (PTX group). A sub-analysis of patients with a severe HPTS, defined as baseline PTH levels > 200 pg/mL and/or serum ionized calcium > 6.0 mg/dL was performed.

Data were collected from electronic charts and included age, sex, and some CKD-MBD laboratory parameters. Serum ionized calcium (iCa; RR = 4.49–5.29 mg/dL) was measured by ion selective electrode. Serum total calcium (TCa; reference range [RR] = 8.4 – 10.2 mg/dL), serum alkaline phosphatase (AP; RR = 35–104 U/L) and serum phosphate (P; RR = 2.7–4.5 mg/dL) were measured using colorimetric assay. Intact parathyroid hormone (PTH; RR 15–65 pg/mL) and serum 25-vitamin D (RR = 30–100 ng/ml) were measured using electrochemiluminescence.

### Statistical Analysis

Data are presented as mean ± SD or median and 25, 75 percentiles, according to distribution. We compared continuous variables between two groups using the student’s t-test or Mann-Whitney U-test, as appropriate. ANOVA or Kruskal-Wallis were applied for comparison among 3 or more groups. The effect of time variation was assessed by repeated measure ANOVA or Friedman test according to data distribution. To compare categorical variables, we used Chi-square or Fisher test, as appropriate. The value of p < 0.05 was determined as statistically significant. We used SPSS 21.0 (SPSS Inc., Chicago IL) and GraphPad Prism 9 Software (GraphPad Software Inc., San Diego, CA, USA) for statistical analyses.

## Results

### CKD 5D Group

**Figure 2.**
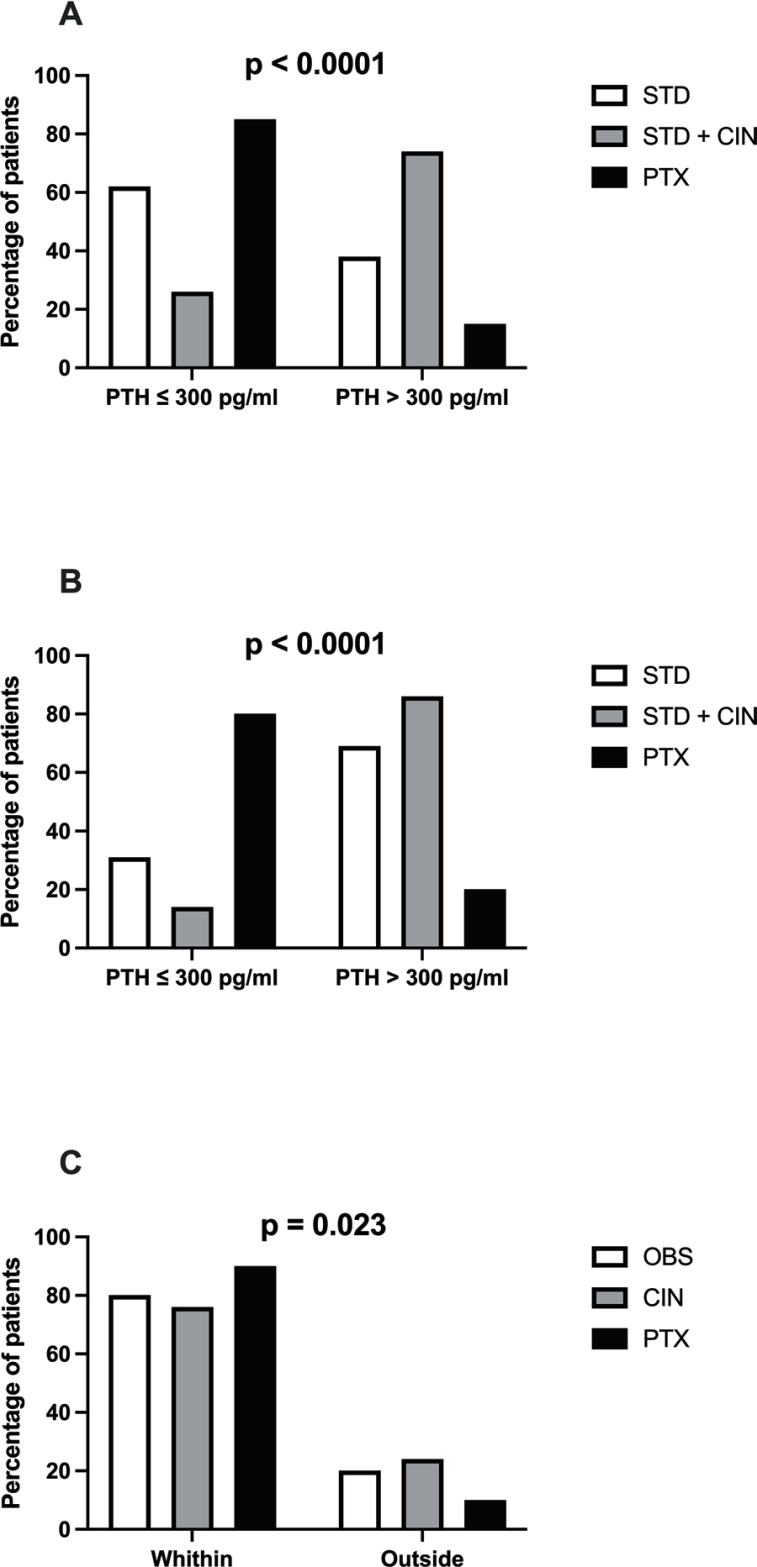
Parathyroid hormone (PTH) control according to the reference range for each group of patients. 2A. Percentage of patients with PTH ≤ or > 300 pg/mL from the standard (STD), standard plus cinacalcet (STD+CIN), and parathyroidectomy (PTX) groups, respectively represented by white, gray, and black bars. 2B. Percentage of patients with severe hyperparathyroidism with PTH ≤ or > 300 pg/mL from the standard (STD), standard plus cinacalcet (STD+CIN), and parathyroidectomy (PTX) groups, respectively indicated in white, gray, and black bars. 2C. Percentage of kidney transplanted patients with PTH/ionized calcium within the normal range (PTH ≤ 100 pg/mL and ionized calcium ≤ 5.3 mg/dl) and outside the normal range in observational (OBS), cinacalcet (CIN), and parathyroidectomy (PTX) groups, respectively represented by white, gray, and black bars.

We observed a significant decrease in serum PTH in the entire cohort (from 996 pg/mL (563;1656) to 473 pg/mL (281;879), p = 0.0001). However, when each group was analyzed separately as shown in Table 1, patients from the CIN group had higher P levels than those under STD therapy, whereas patients from the PTX group had the highest PTH and AP levels. 25-vitamin D levels increased, whereas PTH levels decreased in all groups. Absolute changes (final - initial laboratory values) of PTH and AP levels were greater in patients submitted to PTX. Final PTH was below 300 pg/mL in 62%, 26%, and 85% of patients from STD, STD + CIN, and PTX groups, respectively, at the end of the follow-up period (p < 0.0001, Figure 2A). In patients with severe SHPT, we observed a broader reduction in TCa, iCa, PTH, and AP values in the PTX group compared to the other groups (Table 1). Normal levels of PTH were reached in 31%, 14%, and 80% of patients from STD, STD + CIN, and PTX groups, respectively (p < 0.0001, Figure 2B).

**Table 1:** Characteristics of patients from the CKD group according to treatment

All patients	STD N = 28 baseline	STD N = 28 follow-up	Change	STD + CIN N = 62 baseline	STD + CIN N = 62 follow-up	Change	PTX N = 13 baseline	PTX N = 13 follow-up	Change
Age, years	51 ± 14			51 ± 14			45 ± 13		
TCa, mg/dl	9.3 ± 1.0	9.1 ± 1.1^b,c^	0(–0.2/0.7)	9.2 ± 0.9	9.1 ± 0.9	–0.2(–0.6/0.5)	9.5 ± 1.3	8.5 ± 1.3^a^	–1.0(–1.7/0.1)^d^
iCa, mg/dL	4.77 ± 0.49	4.72 ± 0.58^a^	–0.10 (–0.28/0.29)	4.79 ± 0.51	4.71 ± 0.68	0(–0.37/0.30)	4.83 ± 0.62	4.43 ± 0.65	–0.37 (–1.07/0.29)^d^
P, mg/dL	4.7 ± 1.5^c^	4.8 ± 1.2	0(–0.7/1.0)^c^	5.3 ± 1.6	4.7 ± 1.3^a^	–0.8(–1.6/0.3)	3.8 ± 1.9	4.5 ± 1.9	–0.9(–0.9/1.5)
AP, U/L	129 (98/272)^c^	130 (99/210)^a,c^	1(–33/36)^b^	157 (99/350)^d^	140 (92/359)^a^	–1(–48/31)^b^	532 (360/628)^d^	161 (84/ 282)^a^	–413 (–448/–109)^d^
PTH, pg/mL	825 (409/1,692)	390 (267/671)^a^	–141 (–1,063/80)^b^	880 (560/1,621)	663 (338/1,237)^a,d^	–217 (–528/44)^b^	1,587 (573/2,250)^d^	43 (28/142)^a^	–1,456 (–2,107/–506)^d^
Vit.D, ng/mL	27.8 ± 10.0	31.9 ± 13.4^a^	1(–3.8/10.6)	28.4 ± 10.6	30.2 ± 10.8^a,d^	2.7(–3.6/9.7)	30.6 ± 12.6	40.3 ± 12.9^a^	7.2(0/20.8)
**Severe SHPT**	**STD** **N = 12** **baseline**	**STD** **N = 12** **follow-up**	**Change**	**STD + CIN** **N = 35** **baseline**	**STD + CIN** **N = 35** **follow-up**	**Change**	**PTX** **N = 10** **baseline**	**PTX** **N = 10** **follow-up**	**Change**
Age, years	50 ± 15			47 ± 15			43 ± 14		
TCa, mg/dl	9.6 ± 0.6	9.3 ± 1.2	–0.1(–0.9/0)	9.2 ± 1.1	9.1 ± 0.9	–0.2(–0.7/0.5)^b^	9.7 ± 1.5	8.5 ± 1.4^a^	–1.1(–2.2/–0.1)^d^
iCa, mg/dL	4.85 ± 0.40	4.80 ± 0.64	–0.23(–0.32/0.35)	4.72 ± 0.57	4.61 ± 0.81	–0.01(–0.41/0.34)	4.88 ± 0.69	4.36 ± 0.65	–0.42(–1.17/0.13)^d^
P, mg/dL	5.4 ± 1.3^b^	4.9 ± 1.1	–0.2(–1.3/0.9)	5.9 ± 1.4^b^	4.9 ± 1.4^b^	–0.8(–2.2/–1.0)	4.0 ± 2.0	4.8 ± 2.2	–0.8(–1.8/2.0)
AP, U/L	177 (114/523)	109 (99/401)	–17 (–83/26)	266 (109/388)	217 (99/441)	–1.0(–45/63)^b^	535 (507/808)	205 (84/302)^a^	–420 (–457/–197)^d^
PTH, pg/mL	1,663 (1,078/2,140)	426 (244/1,556)^a^	–439 (–1777/90)	1,394 (1,035/2,020)	856 (554/1,626)^a^	–451 (–694/–140)^b^	1,754 (1,368/2,644)	41(29/136)^a,d^	–1,597 (–2,579/–1,120)^d^
Vit.D, ng/mL	28.3 ± 8.8	29.9 ± 10.8^b^	1.3(–4.0/11.3)	25.8 ± 9.6	27.4 ± 10.3^a^	2.5(–4.3/10.9)	29.6 ± 12.6	38.1 ± 13.7	2.1(–0.2/20.3)

TCa: total calcium; iCa: ionized calcium; P: phosphate; AP: alkaline phosphatase; PTH: parathyroid hormone; Vit:D. 25(OH)-vitamin D. ^a^p < 0.05 vs. baseline in the same group; In the same time point evaluation: ^b^p < 0.05 vs. PTX group; ^c^p < 0.05 vs. cinacalcet; ^d^p < 0.05 vs. all.

### Kidney Transplant Group

There was a reduction in PTH levels in the entire group from a median 153 pg/mL (85; 303) to 29 pg/mL (24;36), p < 0.0001. However, as shown in Table 2, patients from the OBS group presented the lowest TCa and iCa at baseline, whereas patients from the PTX group had the highest iCa and lowest P at the same time point. During the follow-up, absolute changes in PTH and AP were similar among groups, whereas changes in iCa and TCa were larger in the PTX group. Final iCa was higher amongst cinacalcet users compared to the other 2 groups. At the end of the follow-up period, 80% of patients from OBS, 76% of patients from CIN, and 90% of those from the PTX group had PTH and iCa within the normal range (p = 0.023, Figure 2C). No significant difference was seen in graft function in any group. All patients with severe SHPT experienced a reduction in PTH levels. However, a more significant change in TCa, iCa, and P was seen in those who underwent PTX.

**Table 2:** Characteristics of patients from the KTx group according to treatment

All patients	OBS N = 31 baseline	OBS N = 31 follow-up	Change	CIN N = 36 baseline	CIN N = 36 follow-up	Change	PTX N = 10 baseline	PTX N = 10 follow-up	Change
Age, years	49 ± 12			49 ± 14			52 ± 7		
eGFR	55(36, 77)	50(26, 68)	–0.5(–3.6, 5.2)	43(33, 58)	47(34, 59)	0.5(–6.4, 4.2)	55(36, 77)	50(26, 68)	–0.5(–3.6, 5.2)
TCa, mg/dl	9.7 ± 1.0^d^	9.4 ± 0.9^a^	–0.2(–0.7/0.1)	10.3 ± 1.7	9.8 ± 0.9^b^	–0.5(–1.3/0)	10.8 ± 1.1	9.0 ± 0.9^a,d^	–1.8(–2.2/–1)^d^
iCa, mg/dL	5.21 ± 0.62^d^	4.97 ± 0.50^a^	–0.05(–0.38/0.03)	5.60 ± 0.50	5.31 ± 0.46^a,d^	–0.34(–0.66/0.13)	5.96 ± 0.57^d^	4.91 ± 0.47^a^	–0.95(–1.31/–0.65)^d^
P, mg/dL	3.4 ± 0.9	3.4 ± 0.8	0(–0.6/0.5)^d^	3.4 ± 1.9	3.5 ± 1.8	0.3(–0.2/0.7)^d^	2.2 ± 0.6^d^	3.5 ± 1.3	1.9(0.6/3.2)^d^
AP, U/L	85(64/124)^c^	80(63/116)^a,c^	–5(–20/2.0)	113(76/181)	105(75/139)	–2(–59/14)	112(76/216)	72(62/103)^a,c^	–22(–35/5)
PTH, pg/mL	95(53/154)	30(22/37)^a^	–58(–131/–29)	134(75/188)	25(22/34)^a^	–104(–153/–48)	99(32/349)	27(23/33)^a^	–102(–340/–3)
Vit.D, ng/mL	28.9 ± 10.4	29.3 ± 9.1	4,4(–9.5/9.7)	25.4 ± 9.0	28.6 ± 10.4^a^	3.1(–2.9/15.1)	25.9 ± 11.2	34.3 ± 15.5	1.4(–8.3/23.7)
**Severe e SHPT**	**OBS** **N = 6** **baseline**	**OBS** **N = 6** **follow-up**	**Change**	**CIN** **N=18** **baseline**	**CIN** **N = 18** **follow-up**	**Change**	**PTX** **N = 6** **baseline**	**PTX** **N = 6** **follow-up**	**Change**
Age, years	49 ± 12			42 ± 15			50 ± 6		
eGFR	36(35, 75)	37(34, 83)	4(–2, 8)	49(40, 60)	49(41, 56)	0(–4, 5)	45(34, 51)	45(29, 52)	0(–13, 4.5)
TCa, mg/dl	10.1 ± 0.5	10.5 ± 1.4	0.7(0.1/1.2)	11.3 ± 0.7	10.8 ± 0.6^a,b^	–0.5(–0.9/0.5)	11.2 ± 1.3	9.0 ± 1.1	–1.8(–3.2/–1.0)^d^
iCa, mg/dL	5.54 ± 0.39	5.42 ± 0.26^c^	–0.23(–0.31/0.55)	5.86 ± 0.57	5.72 ± 0.31^d^	–0.36(–0.55/0.43)	6.17 ± 0.66	5.00 ± 0.52^c^	–0.95(–1.82–0.65)^d^
P, mg/dL	3.1 ± 0.5	2.8 ± 0.7	–0.2(–0.7/1.5)	3.4 ± 2.1	2.9 ± 1.4	–0.1(–0.6/0.7)	2.3 ± 0.6	4.1 ± 1.4	1.9(0.5/3.3)^d^
AP, U/L	87(52/256)	85(55/263)^c^	–8(–101/35)	122(66/139)	111(59/182)	–4(–30/18)	112(86/247)	86(59/130)	–24(–90/–7)
PTH, pg/mL	75(38/126)	31(22/36)^a^	–29(–83/–0.5)	153(50/949)	26(23/41)^a^	–137(–909/–26)	312(55/573)	27(22/39)^a^	–307(–707/–122)
Vit.D, ng/mL	32.4 ± 8.5	26.9 ± 9.0	–6.4(–6.7/12.9)	32.4 ± 8.5	25.9 ± 8.1	12.3(–1.7/16.7)	26.7 ± 13.7	38.2 ± 19.1	20.3(–15.0/26.7)

TCa: total calcium; iCa: ionized calcium; P: phosphate; AP: alkaline phosphatase; PTH: parathyroid hormone; Vit.D: 25(OH)-vitamin D.

a p < 0.05 vs. baseline; In the same time point evaluation: ^b^p < 0.05 vs. PTX group; ^c^p < 0.05 vs. cinacalcet; ^d^p < 0.05 vs. all.

## Discussion

In most patients in our cohort, whether CKD or KTx, PTH levels were successfully controlled. However, PTX was associated with a greater chance of success. Moreover, this difference in favor of PTX was even more evident when we analyzed only patients with severe forms of SHPT.

SHPT management is known to be challenging, and several factors could be related to therapeutic failure, such as poor adherence to medications and diet, dialysis quality, frequency of PTH monitoring, and timing of treatment initiation. As a result, PTX is frequently adopted as the definitive therapy, with rates of more than 11 procedures per 1,000 patients per year in the 1990s^8^.

The introduction of calcimimetics in 2004 has revolutionized the management of SHPT, leading to changes in indications and appropriate timing for PTX surgery around the world. The number of PTX drastically declined as reported by US^9^, Canadian^10^, European and Japanese groups^5,11^. However, in the US, these rates have increased again, suggesting that in some countries the adoption of more liberal targets for PTH might be associated with the development of more severe forms of SHPT^9^. CKD patients with severe SHPT are generally refractory to medical therapy and usually require surgical PTX, although this is still controversial. Few studies, primarily conducted in Asia, Eastern Europe, and North America, have demonstrated the salutary effects of cinacalcet in lowering PTH levels in severe SHPT^12-14^. However, real-world studies have shown that patients with severe HPTS usually do not respond to clinical management. The MIMOSA study, in France, showed that half of the patients with serum PTH > 1,000 pg/mL still had uncontrolled PTH after a 1-year follow-up^15^. Another concern regarding persistent SHPT in KTx patients, which affects more than 40% of transplant recipients, is that the persistence of hyperparathyroidism for more than one year may be a risk factor for graft failure^13,16^.

In Brazil, despite a growing incident and prevalence of dialysis patients, there is no broad access to CKD-MBD drugs. Until 2022, patients in the public health system were not allowed to receive cinacalcet unless they had a serum PTH higher than 800 pg/mL or persistent hypercalcemia or hyperphosphatemia and a documented failure to achieve adequate PTH levels with VDRAs^17^. Consequently, in 2018, only 11% of the 133,464 patients on dialysis were receiving cinacalcet, whereas 29% and 6% were taking calcitriol and paricalcitol, respectively. This limitation is not seen for drugs usually prescribed to control anemia, with 77% and 50% receiving erythropoietin and intravenous iron, respectively. In this context, the finding of more than 18% of patients with a PTH higher than 600 pg/mL in the same census is no surprise^18^. The perfect storm arises from limited access to parathyroidectomy, leaving hundreds of patients on waiting lists for surgery^19^. These patients are usually referred to CKD-MBD centers, where nephrologists try to manage their PTH while they wait for surgery. Therefore, the results of this retrospective study reflect the inadequate national management of SHPT.

Regarding KTx patients, persistent hyper-parathyroidism is associated with higher rates of renal allograft failure^20^. In Brazil, more than half of the patients submitted to KTx are classified as having severe SHPT^16^.

Our study has some limitations, including its retrospective nature, the small sample size, the heterogeneity of the groups, the lack of medication adherence assessment, and the short follow-up period. In addition, the definition of persistent and severe hyperparathyroidism was somehow arbitrary. This was supported by recent studies^21,22^ that pointed the lack of clear recommendations and optimal PTH targets or indications and timing of PTX. However, these limitations are counterbalanced by study strengths. This is the first study published to date that have enrolled patients from South America with different ethnic and socio-economic background than populations studied by other groups. Although the patients in each group were not similar, this imbalance would favor the STD and STD + CIN groups, as they had lower PTH levels at baseline. Nevertheless, PTX proved to be a more effective treatment.

## Conclusion

We compared cinacalcet and PTX to the minimal standard of care in both CKD and KTx patients and found a clear advantage for the surgical therapy strategy. Despite the therapeutic advances made in the last 20 years, PTX still seems to be the best choice for the treatment of severe secondary hyperparathyroidism, particularly in patients with a longer disease duration and deprived of medical options in the earlier stages.

## References

[1] Kidney Disease: Improving Global Outcomes (KDIGO) CKD-MBD Update
Work Group. CKDMBDUWG. (2017). KDIGO 2017 Clinical Practice Guideline Update for the Diagnosis,
Evaluation, Prevention, and Treatment of Chronic Kidney Disease-Mineral and
Bone Disorder (CKD-MBD).. Kidney Int Suppl (2011)..

[2] Bucharles SGE, Barreto FC, Riella MC (2019). The impact of cinacalcet in the mineral metabolism markers of
patients on dialysis with severe secondary
hyperparathyroidism.. J Bras Nefrol..

[3] Cunningham J, Locatelli F, Rodriguez M (2011). Secondary hyperparathyroidism: pathogenesis, disease progression,
and therapeutic options.. Clin J Am Soc Nephrol..

[4] Bouquegneau A, Salam S, Delanaye P, Eastell R, Khwaja A (2016). Bone Disease after Kidney Transplantation.. Clin J Am Soc Nephrol..

[5] Greeviroj P, Kitrungphaiboon T, Katavetin P, Praditpornsilpa K, Eiam-Ong S, Jaber BL (2018). Cinacalcet for Treatment of Chronic Kidney Disease-Mineral and
Bone Disorder: a meta-analysis of randomized controlled
trials.. Nephron..

[6] Eidman KE, Wetmore JB (2017). The role of parathyroidectomy in the management of secondary
hyperparathyroidism.. Curr Opin Nephrol Hypertens..

[7] Oliveira RB, Silva EN, Charpinel DM, Gueiros JE, Neves CL (2011). Sampaio Ede A, et al. Secondary hyperparathyroidism status in
Brazil: brazilian census of parathyroidectomy.. J Bras Nefrol..

[8] Kestenbaum B, Seliger SL, Gillen DL, Wasse H, Young B, Sherrard DJ (2004). Parathyroidectomy rates among United States dialysis patients:
1990–1999.. Kidney Int..

[9] Kim SM, Long J, Montez-Rath ME, Leonard MB, Norton JA, Chertow GM (2016). Rates and Outcomes of Parathyroidectomy for Secondary
Hyperparathyroidism in the United States.. Clin J Am Soc Nephrol..

[10] Lafrance JP, Cardinal H, Leblanc M, Madore F, Pichette V, Roy L (2013). Effect of cinacalcet availability and formulary listing on
parathyroidectomy rate trends.. BMC Nephrol..

[11] Komaba H, Taniguchi M, Wada A, Iseki K, Tsubakihara Y, Fukagawa M (2015). Parathyroidectomy and survival among Japanese hemodialysis
patients with secondary hyperparathyroidism.. Kidney Int..

[12] Susantitaphong P, Vadcharavivad S, Susomboon T, Singhan W, Dumrongpisutikul N, Jakchairoongruang K (2019). The effectiveness of cinacalcet: a randomized, open label study
in chronic hemodialysis patients with severe secondary
hyperparathyroidism.. Ren Fail..

[13] Mogl MT, Skachko T, Dobrindt EM, Reinke P, Bures C, Pratschke J (2021). Surgery for renal hyperparathyroidism in the era of cinacalcet: a
single-center experience.. Scand J Surg..

[14] Raggi P, Chertow GM, Torres PU, Csiky B, Naso A, Nossuli K (2011). The ADVANCE study: a randomized study to evaluate the effects of
cinacalcet plus low-dose vitamin D on vascular calcification in patients on
hemodialysis.. Nephrol Dial Transplant..

[15] Rottembourg J, Urena-Torres P, Toledano D, Gueutin V, Hamani A, Coldefy O (2019). Factors associated with parathyroid hormone control in
haemodialysis patients with secondary hyperparathyroidism treated with
cinacalcet in real-world clinical practice: mimosa study.. Clin Kidney J..

[16] Araujo M, Ramalho JAM, Elias RM, Jorgetti V, Nahas W, Custodio M (2018). Persistent hyperparathyroidism as a risk factor for long-term
graft failure: the need to discuss indication for
parathyroidectomy.. Surgery..

[17] Brasil. Ministério da Saúde. Portaria nº 48, de 20 de janeiro de
2015. (2015). Habilita os entes federativos ao recebimento do incentivo financeiro de
custeio para implantação e manutenção de ações e serviços públicos
estratégicos de Vigilância em Saúde..

[18] Neves P, Sesso RCC, Thome FS, Lugon JR, Nasicmento MM (2020). Brazilian Dialysis Census: analysis of data from the 2009–2018
decade.. J Bras Nefrol..

[19] Goldenstein PT, Elias RM, Pires de Freitas do Carmo L, Coelho FO, Magalhães LP, Antunes GL (2013). Parathyroidectomy improves survival in patients with severe
hyperparathyroidism: a comparative study.. PLoS One..

[20] Finnerty BM, Chan TW, Jones G, Khader T, Moore M, Gray KD (2019). Parathyroidectomy versus cinacalcet in the management of tertiary
hyperparathyroidism: surgery improves renal transplant allograft
survival.. Surgery..

[21] Cianciolo G, Tondolo F, Barbuto S, Angelini A, Ferrara F, Iacovella F (2022). A roadmap to parathyroidectomy for kidney transplant
candidates.. Clin Kidney J..

[22] Walkenhorst Z, Maskin A, Westphal S, Fingeret AL (2023). Factors associated with persistent post-transplant
hyperparathyroidism after Index Renal Transplantation.. J Surg Res..

